# Health-related quality of life compared between kidney transplantation and nocturnal hemodialysis

**DOI:** 10.1371/journal.pone.0204405

**Published:** 2018-09-20

**Authors:** Thijs T. Jansz, Anna A. Bonenkamp, Franciscus T. J. Boereboom, Franka E. van Reekum, Marianne C. Verhaar, Brigit C. van Jaarsveld

**Affiliations:** 1 Department of Nephrology and Hypertension, University Medical Center Utrecht, Utrecht University, Utrecht, the Netherlands; 2 Department of Nephrology, VU University Medical Center, Amsterdam, the Netherlands; 3 Dianet Dialysis Centers, Utrecht, the Netherlands; 4 Diapriva Dialysis Center, Amsterdam, the Netherlands; Hospital Universitario de la Princesa, SPAIN

## Abstract

**Background:**

Health-related quality of life (HRQOL) is an important outcome measure in patients with end-stage renal disease. HRQOL is assumed to improve with kidney transplantation and also with nocturnal hemodialysis compared to conventional hemodialysis. However, there is no evidence regarding HRQOL to support the optimal treatment choice for patients on nocturnal hemodialysis who hesitate opting for transplantation. We therefore compared HRQOL between patients who were treated with kidney transplantation or nocturnal hemodialysis for one year.

**Methods:**

We assessed HQROL using the Kidney Disease Quality of Life–Short Form questionnaire in a cross-sectional sample of patients who were treated with kidney transplantation *(n = 41)* or nocturnal hemodialysis *(n = 31)* for one year. All patients on nocturnal hemodialysis were transplantation candidates. Using linear regression, we compared HRQOL between kidney transplantation and nocturnal hemodialysis, and adjusted for age, sex, dialysis duration, cardiovascular disease, and presence of residual urine production.

**Results:**

At one year follow-up, mean age of the study population was 54 ±13 years, and median dialysis duration was 3.2 (IQR 2.1–5.0) years. Kidney transplantation was associated with significantly higher HRQOL on the domain “effects” compared to nocturnal hemodialysis (adjusted difference 12.0 points, 95% CI 3.9; 20.1). There were potentially clinically relevant differences between kidney transplantation and nocturnal hemodialysis on the domains “burden” (adjusted difference 11.1 points, 95% CI -2.6; 24.8), “social support” (adjusted difference 6.2, 95% CI -6.6; 19.1), and the physical composite score (adjusted difference 3.0, 95% CI -2.0; 8.1), but these were not significant.

**Conclusions:**

After kidney transplantation, HRQOL is especially higher on the domain “effects of kidney disease” compared to nocturnal hemodialysis. This can be useful when counseling patients on nocturnal hemodialysis who may opt for transplantation.

## Introduction

Health-related quality of life is an important indicator of well-being in patients with end-stage renal disease and is associated with survival and clinical outcomes[1–4]. Compared to the general population, patients with end-stage renal disease have severely diminished health-related quality of life, by some deemed even lower than in diseases such as congestive heart failure, chronic lung disease or cancer[5].

The preferred treatment for end-stage renal disease is kidney transplantation, which is associated with improved health-related quality of life and survival[6]. However, because of the limited availability of donor kidneys and because of transplant failure, many patients have to remain on dialysis.

An alternative to conventional dialysis modalities is frequent nocturnal hemodialysis. With this treatment, patients dialyze almost daily and twice as long (7–8 hours), generally at home. Thus, this treatment removes fluid more slowly and clears more solutes such as urea and phosphate[7]. Nocturnal hemodialysis may hence improve intermediate outcomes[8, 9] and possibly even survival, although mortality data remain inconsistent[10, 11]. By dialyzing at night, patients save time during the day, and nocturnal hemodialysis has thus been reported to improve health-related quality of life[12–14], to such an extent that some patients may even choose to forgo transplantation[15].

How clinicians should deal with this reluctance toward transplantation is unclear. Currently, there is no evidence to support the optimal treatment choice for these patients, particularly not regarding patient-reported outcome measures. To fill this gap, we compared health-related quality of life measured with the Kidney Disease Quality of Life—Short Form (KDQOL-SF) between kidney transplant recipients and transplantation-eligible patients treated with nocturnal hemodialysis.

## Methods

### Study population

We analyzed a cross-sectional cohort from the ongoing NOCTx study (NCT00950573), a prospective cohort study designed to compare progression of coronary artery calcification between kidney transplant recipients, patients on frequent nocturnal home hemodialysis, and patients on chronic peritoneal dialysis or conventional hemodialysis. Patients were eligible when aged between 18 and 75 years and were candidates for transplantation when on dialysis. All study participants gave written informed consent. NOCTx excluded patients with a life expectancy <3 months, pre-emptive transplantation, or non-adherence to dialysis regimens. NOCTx has been approved by the Medical Ethics Committee of the University Medical Center Utrecht and is conducted in accordance with the Declaration of Helsinki.

Between December 2009 and February 2016, NOCTx included 54 kidney transplant recipients and 39 patients on nocturnal hemodialysis who were referred for study participation to the University Medical Center of Utrecht, the Netherlands. For the present analyses, we included all kidney transplant recipients (*n = 41*) and patients on nocturnal hemodialysis (*n = 31*) who had one-year follow-up data. Most patients with a kidney transplant and on nocturnal hemodialysis entered NOCTx 2–3 months after switching to their respective treatment; thus, data from before switching were not available in these patients. We therefore analyzed data cross-sectionally after one year of treatment.

### Treatment characteristics

Patients received treatment according to guidelines by the attending nephrologists. Kidney transplant recipients were treated in two tertiary centers, where standard immunosuppressant regimens consisted of a calcineurin inhibitor (tacrolimus), mycophenolate mofetil, and prednisone in tapering doses. Patients on nocturnal hemodialysis were trained and monitored in two dialysis centers that offered specialized training programs for nocturnal home hemodialysis. Patients dialyzed ≥ 4 x 8 hours per week at home, on a single needle, with a lower effective blood flow (150–220 mL/min), lower dialysate flow (300 mL/min), and a somewhat lower bicarbonate concentration compared to conventional hemodialysis, which was adjusted depending on laboratory results. Unfractionated heparin was used as anticoagulation.

### Health-related quality of life

We assessed health-related quality of life with the validated KDQOL-SF version 1.2[16]. The KDQOL-SF consists of a general part and a disease-specific part. The general part, the Short Form with 36 questions (SF-36) version 1[17], consists of eight domains that can be summarized in two scores. These summary scores are designed to reflect the general population in the United States when the means are 50 with a standard deviation of 10 points for physical functioning (physical composite score) and mental functioning (mental composite score)[18]. The composite scores were obtained from 12 questions in the SF-36 (PCS-12 and MCS-12)[1]. The disease-specific part of the KDQOL-SF consists of 44 kidney disease-targeted questions, grouped in 12 domains. We focused on the domains “symptoms of kidney disease”, “effects of kidney disease”, “burden of kidney disease”, “cognitive function”, “quality of social interaction”, “sexual function”, “sleep”, “social support” and “overall health”. We did not evaluate the domains “work status”, “patient satisfaction” and “dialysis staff encouragement” in this study. The domains are scored from 0 to 100, with higher scores indicating better quality of life. Explanations of the disease-specific domains are available as Table 1 (adapted from Carmichael et al.[19]).

**Table 1:** Explanation of the Kidney Disease Quality of Life-Short Form (KDQOL-SF) kidney disease-specific domains.

Domains	Interpretation	
	Low score	High score
Symptoms of kidney disease	Extremely bothered by dialysis-related symptoms such as muscle cramps, pruritus, anorexia, and/or access problems	Not at all bothered
Effect of kidney disease on daily life	Extremely bothered by fluid and dietary restriction, by an inability to travel, and dependency on doctors	Not at all bothered
Burden of kidney disease	Extremely bothered by the time consumed by dialysis, its intrusiveness, and degree burden on family	Not at all bothered
Cognitive function	Affected all of the time by inability to concentrate, confused, with poor reaction time	Not at all affected
Quality of social interaction	Continual irritation and failure to get along with people with virtual isolation	No problems, socially interactive
Sexual function	Experiencing severe problems with enjoyment and arousal	No problems
Sleep	Very poor sleep with daytime somnolence	No problems with sleep
Social support	Very dissatisfied	Satisfied with level of social support
Overall health	Rates health as worst possible	Rates health as best possible

Adapted from Carmichael et al.[19].

### Other variables

At time of questionnaire completion, study personnel recorded demographical and clinical parameters (pre-dialysis blood pressure and post-dialysis weight averaged from routine measurements during 3 hemodialysis sessions or 2 outpatient visits for kidney transplant recipients) and laboratory parameters (total calcium, phosphate, parathyroid hormone, total cholesterol, albumin, hemoglobin, and C-reactive protein) routinely measured at local treatment facilities. Study personnel assessed presence of comorbidities by chart review, and assessed residual urine production with the most recent 24h-urine collection, which we classified as present (≥100mL/24u) or absent. Smoking status, oral anticoagulant use, and educational level were self-reported.

We defined diabetes mellitus as use of oral anti-diabetic medication or insulin therapy, and cardiovascular disease as any history of angina, myocardial infarction, percutaneous coronary intervention, coronary artery bypass grafting, stroke, intermittent claudication, peripheral artery angioplasty or bypass grafting. We defined higher education as any tertiary education. We estimated glomerular filtration rate with the Chronic Kidney Disease-Epidemiology Collaboration equation 2009 for kidney transplant recipients.

### Statistical analyses

We reported data as number (proportion) for categorical data, mean ±standard deviation for normally distributed variables, and median (interquartile range [IQR]) for non-normally distributed variables. We presented patient characteristics and health-related quality of life by renal replacement therapy. We compared categorical data with chi-squared tests, normally distributed variables with t-tests, and non-normally distributed variables with Mann-Whitney-U tests.

We used multiple linear regression analyses to examine the associations between renal replacement therapy and health-related quality of life. We regarded 5-point differences clinically relevant in the disease-specific domains, and 3-point differences clinically relevant in the composite scores[17, 18]. We adjusted stepwise for potential confounders age (years), sex, educational level (high/low), dialysis duration (years), presence of diabetes mellitus, cardiovascular disease, and presence of residual urine production (≥100mL/24u or absent), and kept them in the model when coefficients changed >10%. In the final model, we adjusted for age, sex, dialysis duration, cardiovascular disease, and presence of residual urine production.

We reported regression coefficients with 95% confidence intervals (CI). We considered P-values ≤ 0.05 (two-tailed) statistically significant, did not attempt imputation for missing values, and performed all analyses with R 3.4.1[20].

## Results

### Study population

The mean age of the study population *(n = 72)* was 54 ±13 years, 50 (69%) were male, median dialysis duration was 38 (IQR 25–60) months, and 17 (24%) had a history of cardiovascular disease. There were no significant differences in demographics or medical history between the kidney transplant recipients (*n = 41*) and patients on nocturnal hemodialysis (*n = 31*), but kidney transplant recipients had significantly lower phosphate levels and higher hemoglobin levels (Table 2). Kidney transplant recipients had an estimated glomerular filtration rate of 54.8 ±15.7 mL/min, while patients on nocturnal hemodialysis had median 0 (IQR 0–250) mL/day residual urine production. Patients on nocturnal hemodialysis dialyzed 38.3 ±7.2 hours per week in 4.8 ±0.8 sessions per week.

**Table 2:** Characteristics of the 72 kidney transplant recipients and patients on nocturnal hemodialysis at one year of follow-up.

	Kidney transplantation (n = 41)	Nocturnal hemodialysis (n = 31)	*P-value*
***Demographics***			
**Age (yr)**	54.0 ±13.8	53.9 ±12.5	0.97
**Male (%)**	31 (75)	19 (62)	0.29
**Body mass index (kg/m^2^)**	25.5 ±4.2	26.5 ±5.2	0.37
**Systolic blood pressure (mmHg)**	132 ±14	139 ±20	0.11
**Diastolic blood pressure (mmHg)**	80 ±10	75 ±12	0.12
**Current smoker (%)**	6 (15)	6 (19)	0.83
**Oral anticoagulant use (%)**	5 (13)	2 (7)	0.66
**Higher education (%)**	11 (28)	8 (26)	0.99
***Medical history***			
**Dialysis duration (mo)**	28 (24–58)	39 (28–66)	0.12
**End-stage renal disease duration (mo)**	28 (25–62)	39 (28–94)	0.15
**Cause of end-stage renal disease (%)**			0.23
**Glomerulonephritis**	9 (22)	11 (36)	
**Interstitial nephritis**	1 (2)	0 (0)	
**Cystic kidney disease**	14 (34)	5 (16)	
**Renovascular**	9 (22)	3 (10)	
**Diabetes mellitus**	1 (2)	2 (7)	
**Other**	3 (7)	5 (16)	
**Unknown**	4 (10)	5 (16)	
**Comorbidities (%)**			
**Diabetes mellitus**	3 (7)	4 (13)	0.70
**Prior cardiovascular disease**	7 (17)	10 (32)	0.22
***Laboratory parameters***			
**Calcium (mmol/L)**	2.41 ±0.10	2.37 ±0.20	0.30
**Phosphate (mmol/L)**	0.88 ±0.21	1.42 ±0.39	<0.001
**Parathyroid hormone (pmol/L)**	8.5 (6.4–12.0)	13.8 (7.6–22.8)	0.14
**Cholesterol (mmol/L)**	5.0 ±1.1	4.6 ±1.0	0.26
**Albumin (g/L)**	42.4 ±3.1	42.4 ±3.1	0.95
**Hemoglobin (mmol/L)**	8.9 ±1.0	7.0 ±0.8	<0.001
**C-reactive protein (mg/L)**	3.0 (2.0–8.3)	5.0 (3.0–10.0)	0.29

Results are presented as mean ±standard deviation, median (interquartile range), or number (proportion).

The current sample comprised 77% of all kidney transplant recipients and patients on nocturnal hemodialysis who entered NOCTx *(n = 93)*. Seven kidney transplant recipients (3 were lost to follow-up, 2 withdrew consent, 2 died) and 7 patients on nocturnal hemodialysis (3 received a transplant, 2 withdrew consent, 1 was lost to follow-up, 1 died) did not complete follow-up at one year, while 6 kidney transplant recipients and 1 patient on nocturnal hemodialysis did not complete quality of life questionnaires at the one-year follow-up. Their mean age (*n = 21*) was 49 ±14 years (P = 0.15 versus study population), 12 (57%) were male (P = 0.43 versus study population), median dialysis duration was 65 (IQR 42–84) months (P = 0.03 versus study population), and 4 (19%) had a history of cardiovascular disease (P = 0.89 versus study population). Kidney transplant recipients were not more likely to complete follow-up than patients on nocturnal hemodialysis (P = 0.88).

### Health-related quality of life at one year of treatment

The quality of life questionnaires were generally well-completed. In the following scales, one or more questionnaire items were missing resulting in a missing score: “sexual function” (5 respondents, 7%), SF-12 items (physical and mental composite scores; 2 respondents, 3%), “symptoms of kidney disease”, “effects of kidney disease”, “burden of kidney disease”, and “overall health” (1 respondent each, 1%).

**Fig 1:**
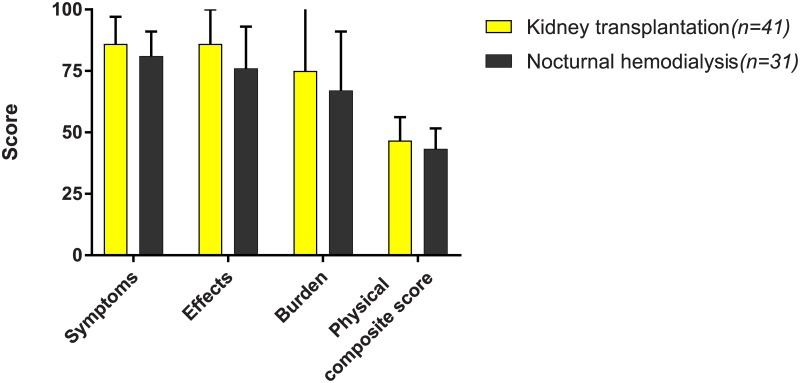
Disease-specific health-related quality of life scores and physical composite scores in the 72 kidney transplant recipients and patients on nocturnal hemodialysis. Mean health-related quality of life scores on the disease-specific domains “symptoms”, “effects”, “burden of kidney disease”, and the physical composite scores as bar charts in the 72 kidney transplant recipients and patients on nocturnal hemodialysis. We presented 95% confidence intervals alongside the bars. Mean scores for kidney transplantation and nocturnal hemodialysis: “symptoms” 86 and 81; “effects” 86 and 76; “burden” 75 and 67; physical composite score 47 and 43 points, respectively.

Overall, kidney transplant recipients had numerically higher scores on the kidney disease-specific domains of health-related quality of life and the physical composite score compared to patients on nocturnal hemodialysis (Fig 1). Kidney transplant recipients scored significantly higher on the domain “effects of kidney disease” compared to patients on nocturnal hemodialysis, both in crude and adjusted analyses (Table 3). There were no significant differences on the other kidney disease-specific domains or the composite scores in both crude and adjusted analyses. When adjusted for age, sex, dialysis duration, cardiovascular disease, and residual urine production, kidney transplant recipients had potentially clinically relevant higher scores on the domains “burden of kidney disease”, “social support”, and the physical composite score compared to nocturnal hemodialysis, but these differences were not significant.

**Table 3:** Health-related quality of life scores and differences in scores between the 72 kidney transplant recipients and patients on nocturnal hemodialysis at one year of follow-up.

	Kidney transplantation (n = 41)	Nocturnal hemodialysis (n = 31)	*Crude difference* *(95% CI)*	Adjusted* *difference* *(95% CI)*
***Kidney disease-related quality of life***				
**Symptoms of kidney disease**	86 ±11	81 ±10	-5.7 (-10.7; -0.7)	-4.6 (-10.6; 1.3)
**Effects of kidney disease**	86 ±14	76 ±17	-9.8 (-16.9; -2.6)	-12.0 (-20.1; -3.9)
**Burden of kidney disease**	75 ±27	67 ±24	-8.0 (-20.1; 4.1)	-11.1 (-24.8; 2.6)
**Cognitive function**	81 ±19	78 ±18	-2.5 (-11.3; 6.3)	-4.3 (-14.2; 5.6)
**Quality of social interaction**	79 ±15	77 ±14	-1.3 (-8.3; 5.8)	1.4 (-6.7; 9.5)
**Sexual function**	72 ±30	64 ±33	-7.8 (-23.1; 7.5)	-2.0 (-19.1; 15.0)
**Sleep**	66 ±23	63 ±16	-2.8 (-12.3; 6.8)	-3.3 (-14.5; 8.0)
**Social support**	87 ±21	82 ±25	-4.7 (-15.5; 6.0)	-6.2 (-19.1; 6.6)
**Overall health**	70 ±16	65 ±17	-4.3 (-12.3; 3.6)	-4.9 (-14.1; 4.3)
***SF-12 composite scores***				
**Physical composite score**	47 ±10	43 ±8	-3.4 (-7.7; 0.9)	-3.0 (-8.1; 2.0)
**Mental composite score**	51 ±10	52 ±11	0.6 (-4.2; 5.5)	1.2 (-4.4; 6.8)

Abbreviations: SF-12: short form-12 items. Scores are presented as mean ±standard deviation, and differences with 95% confidence intervals.

*Adjusted for age (years), sex (male/female), dialysis duration (years), cardiovascular disease, and presence of residual urine production (≥100mL/24u or absent).

## Discussion

To our knowledge, this is the first study to compare health-related quality of life between kidney transplantation and nocturnal hemodialysis, demonstrating that kidney transplantation is associated with significantly higher quality of life on the domain “effects of kidney disease” compared to nocturnal hemodialysis. In addition, kidney transplant recipients have potentially clinically relevant higher quality of life on the domains “burden of kidney disease”, “social support”, and the physical composite score, although not significantly higher in this study. Together, these findings suggest that health-related quality of life is generally better after kidney transplantation than on treatment with nocturnal hemodialysis.

The differences in health-related quality of life are the most evident on the domain “effects of kidney disease”. As this domain involves the restraints patients experience regarding their diet, ability to travel, and dependency on doctors, it is explainable that kidney transplant recipients score higher on this domain. After all, kidney transplant recipients are freer in terms of diet and travel than any patient on dialysis. Besides this domain, kidney transplant recipients have numerically higher adjusted scores on the domains “burden of kidney disease”, “social support”, and the physical composite score. Although not *statistically significant*, these differences may be *clinically relevant*[21]. The original KDQOL-SF manual reads that 5-point differences are clinically relevant regarding the disease-specific domains, and 3-point differences regarding the composite scores[17, 18], which has been adopted by others[22, 23]. Notably, a 3-point difference in the composite scores is associated with a mortality risk of approximately 6.0%[2, 3, 24]. Given the size and consistent direction of these differences, we consider them relevant, even though they do not reach statistical significance with the current sample size.

In our experience, some patients treated with nocturnal hemodialysis decline kidney transplantation and prefer to stay on treatment with nocturnal hemodialysis. The current findings suggest that kidney transplantation—in which quality of life is known to improve[25–27]—is a more favorable treatment option regarding health-related quality of life for transplantation-eligible patients on nocturnal hemodialysis, although individual outcomes may differ importantly.

For patients that are unlikely to receive a kidney transplant (e.g. HLA-sensitized patients), potential benefits of nocturnal hemodialysis remain relevant, such as an improvement of quality of life. Importantly, health-related quality of life has been shown to improve after conversion to nocturnal hemodialysis from conventional hemodialysis in several observational studies[12, 14] and on selected domains in a randomized trial[28]. This is despite the fact that nocturnal hemodialysis is performed almost daily and requires considerable patient involvement. Notably, patients on nocturnal hemodialysis in our cohort have somewhat higher health-related quality of life scores compared to North-American cohorts[12, 13, 29], which may be because all patients were transplantation candidates in our study. Remarkably, nocturnal hemodialysis does not seem to deteriorate sleep quality: patients on nocturnal hemodialysis have similar scores on the domain “sleep” to kidney transplant recipients in our study.

The results of this study should be interpreted within the context of some limitations. First, our study is not powered to demonstrate significance of all potentially relevant differences in kidney disease-specific health-related quality of life domains. For example, we would have needed 161 patients per group to show significance of an 8-point difference (as currently found) in the disease-specific domain “burden of kidney disease”. Second, the current data are cross-sectional after one year of treatment with kidney transplantation or nocturnal hemodialysis. A before/after comparison of health-related quality of life was not possible as patients were included in this study shortly after they had started treatment with either kidney transplantation or nocturnal hemodialysis. Third, we do not know the reasons why individual patients converted to nocturnal hemodialysis–there may have been patient selection. As noted in previous studies, healthier and more motivated patients may have been preferentially selected for nocturnal hemodialysis[30], which could influence health-related quality of life. Also, the current data are observational, although it should be noted that randomization to kidney transplantation would be unethical.

Our study has several strengths. First, questionnaire response rate in this study is high (91%) as compared to large studies on patients on hemodialysis[1, 2]. The responders’ demographic characteristics are largely similar to that of non-responders; therefore, we consider our findings generalizable to patients on nocturnal hemodialysis who may opt for kidney transplantation. Second, we focus on kidney disease-specific domains of health-related quality of life alongside the physical and mental composite scores, which increases the ability to detect more specific differences in patients’ well-being. Finally, this study has only included patients on nocturnal hemodialysis who were transplantation candidates; simultaneously, no kidney transplant recipients had been transplanted pre-emptively, i.e. all recipients had a history of dialysis treatment. Both of these inclusion criteria enable valid comparisons between the treatment groups.

In conclusion, health-related quality of life is higher after kidney transplantation especially on the domain “effects of kidney disease” compared to nocturnal hemodialysis. This can be useful when counseling patients on nocturnal hemodialysis who may opt for transplantation.

## Supporting information

S1 Dataset Source data on which the results of this study are based. (CSV)
